# Prediction-error-dependent processing of immediate and delayed positive feedback

**DOI:** 10.1038/s41598-024-60328-8

**Published:** 2024-04-27

**Authors:** Constanze Weber, Christian Bellebaum

**Affiliations:** Faculty of Mathematics and Natural Sciences, Institute of Experimental Psychology, Department of Biological Psychology, Heinrich Heine University Düsseldorf, Universitätstraße 1, 40255 Düsseldorf, Germany

**Keywords:** Prediction error, Feedback delay, FRN, RewP, Reinforcement learning, Human behaviour, Operant learning, Reward

## Abstract

Learning often involves trial-and-error, i.e. repeating behaviours that lead to desired outcomes, and adjusting behaviour when outcomes do not meet our expectations and thus lead to prediction errors (PEs). PEs have been shown to be reflected in the reward positivity (RewP), an event-related potential (ERP) component between 200 and 350 ms after performance feedback which is linked to striatal processing and assessed via electroencephalography (EEG). Here we show that this is also true for delayed feedback processing, for which a critical role of the hippocampus has been suggested. We found a general reduction of the RewP for delayed feedback, but the PE was similarly reflected in the RewP and the later P300 for immediate and delayed positive feedback, while no effect was found for negative feedback. Our results suggest that, despite processing differences between immediate and delayed feedback, positive PEs drive feedback processing and learning irrespective of delay.

## Introduction

On local trains in Germany, passengers must press a button to open the exit doors when they want to get off. In some trains, the door opens only after a delay of a couple of seconds. Passengers are often irritated by this and keep pressing the button until the doors finally open. This simple observation reveals at least two aspects about goal-directed actions. First, such actions are motivated by an expected outcome, and second, this expectation does not only refer to what is going to happen, but also to when something is going to happen. The neural mechanisms involved in the processing of action outcomes have been studied extensively. In many studies human study participants were given positive or negative feedback for choice actions, often in the form of monetary reward vs non reward or punishment (see e.g., refs.^1–4^, for review see ref.^5^). Rewards are processed in dopamine (DA) neurons in the midbrain^6,7^, which code a reward prediction error (PE) in their firing rate, referring to the difference between the expected and the actually obtained outcome. PE-related information is projected to the striatum as well as to the medial prefrontal and (anterior) cingulate cortex^8^, both belonging to the so-called reward system of the brain^9^. In accordance with the initial example, the neural mechanisms of feedback processing and learning depend on feedback timing. While the striatum is more strongly involved in feedback processing and learning when feedback is given shortly after a choice action (within ca. 2 s), the hippocampus and medial temporal lobe (MTL) play a more important role for processing of and learning from feedback that is delayed by a couple of seconds^10–13^. With the striatum and hippocampus representing qualitatively different types of learning, it has been suggested that immediate feedback drives more implicit/non-declarative learning, while delayed feedback underlies more explicit and declarative learning^14^.

Differences in processing immediate and delayed feedback were also found using electroencephalography (EEG). Feedback has been described to elicit an event-related potential (ERP) component which is more negative for negative compared to positive feedback^15,5^, as is typically revealed by the ERP difference wave between the two feedback types^16^. Originally referred to as feedback(-related) negativity (FRN), it has later been suggested that the pronounced negativity for negative feedback reflects an N200, which is suppressed by a relative positivity for rewards, termed reward positivity (RewP)^17–19^. In line with the assumption that the FRN/RewP reflects a DA-driven reinforcement learning signal^20^, its amplitude has been shown to be modulated by expectancy^1,21–23^ and to scale with the PE^24–27^. This finding as well as results obtained with source analysis techniques and concomitant EEG and fMRI assessments linking its amplitude to processing in the posterior medial frontal cortex/ACC and the striatum^28–30^, suggest that the FRN/RewP can be considered as a neural indicator of PE-driven implicit/non-declarative feedback processing by the reward system. With respect to feedback timing, the amplitude difference between positive and negative feedback is consistently reduced for delayed compared to immediate feedback^2,26, 31–35^. In accordance with the studies described above this finding has been interpreted in terms of reduced involvement of the striatum-based systems for non-declarative learning when learning from delayed feedback (ref.^14^, but see ref.^36^ for an interpretation in terms of temporal predictability).

There are, however, also similarities between the processing of and learning from immediate and delayed feedback. For example, we found that a typical bias for enhanced learning from negative compared to positive feedback induced by DA level reductions in Parkinson’s Disease (PD) patients Off medication^37^ does not only affect learning from immediate, but also from delayed feedback^38^, suggesting a role of striatal DA in feedback learning irrespective of feedback timing. With respect to timing effects on the FRN/RewP, distinguishing between expected and unexpected negative and positive feedback revealed that the negative–positive feedback difference wave has a larger amplitude for unexpected feedback irrespective of feedback delay^31^. This finding might indicate that PE processing in the FRN/RewP is similar for immediate and delayed feedback. Indeed, it seems plausible that the relative contributions of the non-declarative striatum-based and the declarative hippocampus-based systems to feedback processing and learning vary in a graded rather than in an all-or-nothing manner^14^, especially as the two systems have been shown to be able to work together^39^. The previous study examining expectancy effects on the processing of immediate and delayed feedback^31^ entailed, however, only two expectancy levels based on the objective reward probability across experimental trials and applied average-based ERP analyses. This approach neglects trial-by-trial fluctuations and interindividual differences in subjective reward expectations, and, thus, the PEs. With the advent of single-trial-based analyses it has become possible to relate ERP components directly to model-derived latent variables such as the PE^24,40^. And also from a theoretical point of view examining the relationship between ERP components and the PE directly is to be preferred, as the PE has been shown to be reflected in neural activity in many brain structures of the reward system^27,25^. A direct comparison of PE processing in single experimental trials between immediate and delayed feedback has not been conducted so far.

The main aim of the present study was therefore to compare PE processing between immediate (after 1 s) and delayed feedback (after 7 s) by applying reinforcement learning models to derive PE values for each experimental trial in combination with ERP single-trial analyses (see refs.^24–27, 40, 41^ for similar approaches). We used data from a previously published study^32^ which compared immediate and delayed feedback processing in active and observational learning and did not address expectancy or PE effects. We left out the observational learning data in the reanalysis (factor agency), because the focus was on the comparison between immediate and delayed feedback and, more importantly, an estimation of trial-by-trial changes in stimulus values, and thus PEs, by means of a reinforcement learning model requires choice actions of participants in each trial which were not conducted in observational learning. The task applied in the previous study was the same as in ref.^31^, where we found an expectancy effect on the FRN/RewP also for delayed feedback. Based on this finding we hypothesized that the PE would be reflected in the ERP signal in the respective time window for both, immediate and delayed feedback. At the same time, it is conceivable that there is a stronger relationship between the PE and ERP amplitudes in the FRN/RewP time window for immediate than delayed feedback, which would be in line with a stronger involvement of the DA/reward system in immediate feedback processing^14^. A further issue of interest was whether PE processing for immediate and delayed feedback is compatible with the notion that the ERP signal between 200 and 300 ms after feedback presentation is mainly driven by a RewP, that is, by a distinct processing of unpredicted rewards rather than unpredicted feedback in general or unpredicted punishments. A recent study using a time estimation task has shown that the effect of expectedness on the FRN/RewP is stronger for positive feedback^40^, which may suggest that the ERP amplitude in this time window is more strongly affected by the PE for positive feedback. As previous studies examining immediate feedback have also shown that PE processing for positive and negative feedback can vary in terms of latency and spatial distribution^30^, an additional analysis was conducted on a later ERP component that has also been shown to be modulated by feedback-related variables, the P300, which was not addressed in our previous study^32^. The P300 is enhanced for unexpected relative to expected^40^ and for immediate compared to delayed feedback^26,31, 42^. Regarding a modulation by the PE, previous findings are mixed. While a recent meta-analysis^43^ suggested an encoding of a PE and valence also in the P300 time window, albeit considerably smaller than for the FRN/RewP^23^, several single-trial-based studies examining immediate feedback did not find PE effects^24,25^. Finally, we did not analyze theta power in response to feedback, which was analyzed in the previous study, as our focus was on feedback delay effects on the relationship between the PE and specific ERP components, which has been reported in previous studies.

## Results

**Figure 1:**
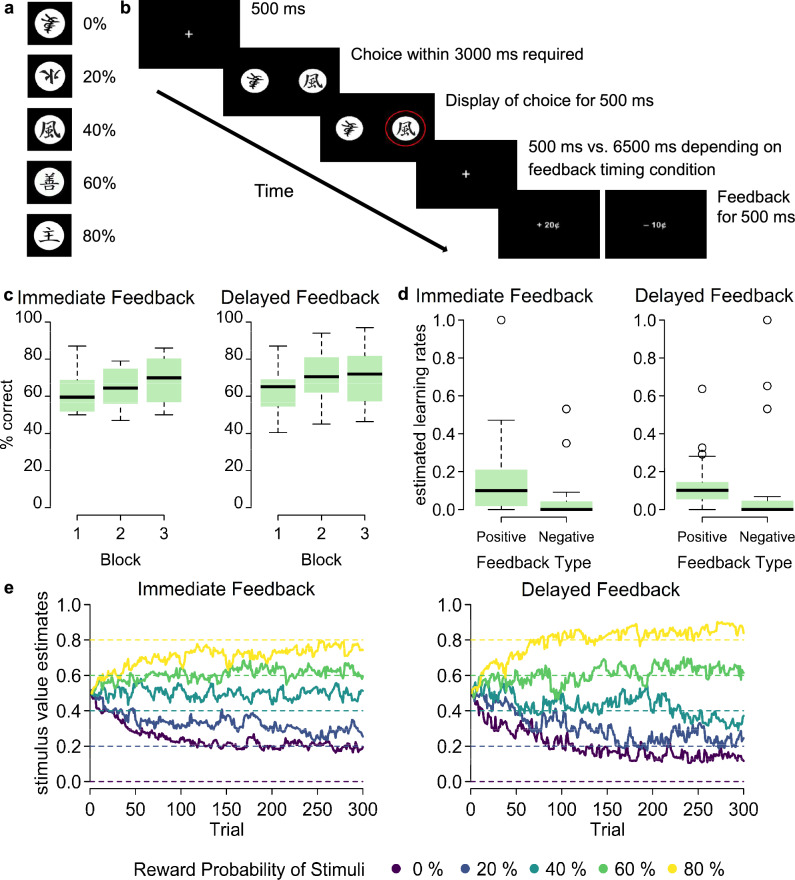
Probabilistic learning task and behavioural parameters. (**a**) Visual stimuli. One of the two sets of symbols used as visual stimuli with their corresponding reward probabilities in the probabilistic learning task. To enable and compare learning in both feedback timing conditions, each participant underwent the task with a different set of visual stimuli in the immediate and delayed feedback version (counterbalanced between participants). (**b**) Schematic trial. The time course of events in a learning trial is shown. Participants’ choice of one of the two presented stimuli was indicated by a red circle for 500 ms after their response, followed by a fixation cross for 500 ms in the immediate feedback condition and for 6500 ms in the delayed feedback timing condition. The feedback was then displayed for 500 ms. Intertrial intervals varied between 1200 and 1600 ms. Participants who did not respond within 3000 ms were asked to respond more quickly. (**c**) Learning performance. Boxplots show averaged choice accuracies across all reward probabilities and participants (N = 20) separately for each block in each feedback timing condition. (**d**) Estimated learning rates. Boxplots show learning rates averaged across participants (N = 20) which were estimated separately for positive and negative feedback trials and each feedback timing condition. (**e**) Stimulus value estimates over the course of the experiment, separately for the five stimuli involved and the immediate and delayed feedback timing condition, averaged across participants (N = 20). Dashed lines show the objective reward probabilities for comparison.

Twenty healthy participants performed the probabilistic learning task with 300 trials (organized in three separate blocks) once with immediate feedback (1 s) after their choice action and once with delayed feedback (7 s) while EEG was recorded^32^. In each trial participants had to select one out of two displayed visual stimuli (randomly drawn from a set of five stimuli, Fig. 1a) and received positive (monetary reward) or negative feedback (monetary punishment), either immediately or delayed (Fig. 1b). Importantly, the five stimuli were assigned different probabilities determining how likely they were to be rewarded when selected (0 vs 20 vs 40 vs 60 vs 80%) such that participants were able to learn, i.e. develop differentiated expectations regarding the outcome when choosing one of the five stimuli over trials.

A repeated-measures ANOVA with choice accuracy as the dependent variable (choices were regarded as correct when the stimulus with the higher reward probability was selected) and the two within-subjects factors Learning Block (1–3) and Feedback Timing (immediate, delayed) revealed a significant main effect of Learning Block, *F*(1.14, 21.74) = 9.56, *p* = 0.004, η_p_^2^ = 0.062. Separate paired t-tests for the comparison of performance between blocks revealed a significant increase in accuracy between the first and the second, *t*(39) = − 3.64, *p* = 0.002, the first and the third, *t*(39) = − 4.08, *p* = 0.001, but not the second and the third learning block, *t*(39) = − 2.18, *p* = 0.106, confirming that participants indeed learned which stimuli were associated with a lower/higher probability to be rewarded over the course of the experiment (Fig. 1c). Neither the main effect of Feedback Timing, *F*(1, 19) = 1.17, *p* = 0.293, η_p_^2^ = 0.012, nor the interaction between Learning Block and Feedback Timing,* F*(2, 38) = 2.06, *p* = 0.142, η_p_^2^ = 0.005, reached significance, indicating that learning performance did not differ between the immediate and delayed feedback timing condition.

Based on the participants’ sequence of choices and the respective feedback, we modelled learning rates (Fig. 1d) and single-trial stimulus values (Fig. 1e), i.e. latent expectations regarding the five stimuli for each trial, for each participant and separately for the immediate and delayed feedback timing condition using a standard reinforcement learning model (see ‘Methods’ for details of model specification and selection). Learning rates, estimated separately for positive and negative feedback, indicate to which degree the PE, that is, for each trial the difference between current stimulus value (expectation) and feedback (outcome), is used to update stimulus values for subsequent trials. As depicted in Fig. 1d, learning rates for negative feedback converge to a value proximate to 0 (with a median of 10^−10^), regardless of feedback delay. This indicates that learning was driven primarily by positive rather than negative feedback. A Wilcoxon signed-rank test comparing learning rates between Feedback Type (positive vs negative) confirmed that learning rates were significantly larger for positive feedback (*Mdn* = 0.1) than for negative feedback (*Mdn* = 0), Z = 3.49, *p* < 0.001, *r* = 0.78. Thus, in line with a choice-confirmation bias^44^, stimulus value updates were driven more strongly by PEs after positive feedback, that is, in trials in which the participants’ choices were confirmed, compared to trials with negative, disconfirmatory feedback. A Wilcoxon signed-rank test comparing learning rates between Feedback Timing (immediate vs delayed) showed that learning rates did not differ significantly between immediate (*Mdn* = 0.03) and delayed feedback (*Mdn* = 0.05), Z = − 0.31, *p* = 0.765,* r* = − 0.07, indicating that the degree to which the PE was used to update the stimulus value did not differ between the immediate and delayed feedback timing condition. To further test whether feedback timing differentially affected learning rates for the different Feedback Types, a Wilcoxon signed-rank test compared the difference between learning rates for positive and negative feedback between the immediate (*Mdn* = 0.08) and delayed (*Mdn* = 0.09) feedback timing condition. This comparison did not yield a significant difference, Z = 0.56, *p* = 0.596, *r* = 0.13.

### FRN/RewP and model-derived trial-level PE

**Figure 2:**
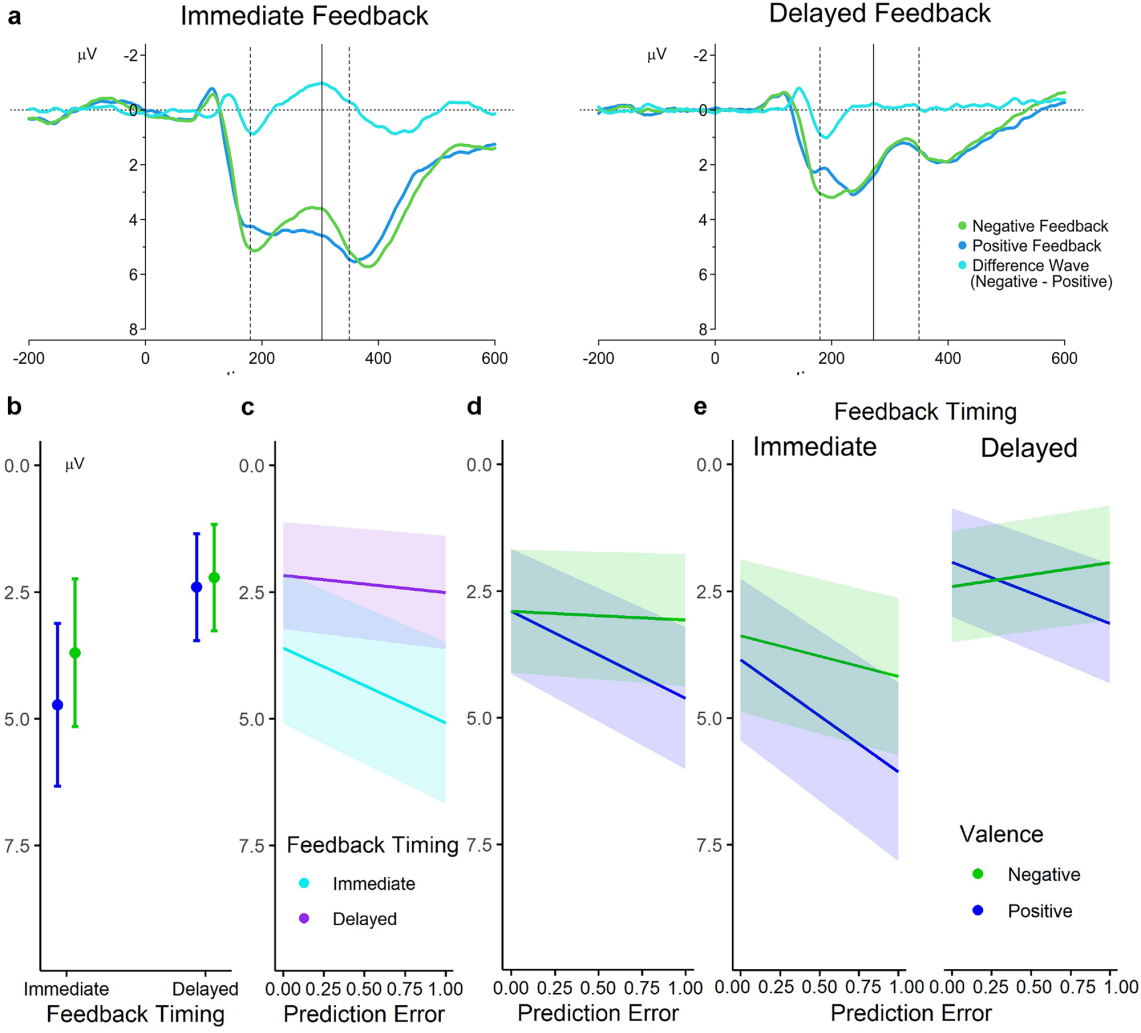
FRN/RewP quantification and results. (**a**) Feedback-locked grand-averaged ERPs at the frontocentral electrode cluster separately for the immediate and delayed feedback timing condition. Dashed lines show the time window in which the FRN/RewP was quantified. The peak latency of the difference wave (negative minus positive feedback) on which the time window for amplitude extraction was based is shown with the solid line. (**b**) Model-estimated marginal effects illustrating the interaction between the fixed effects of Feedback Valence and Feedback Timing, (**c**) the interaction between the fixed effects of PE and Feedback Timing, and (**d**) the interaction between the fixed effects of PE and Feedback Valence (regardless of Feedback Timing). In (**e**), the exploratory follow-up analyses of the interaction between PE and Feedback Valence separately for the immediate and delayed Feedback Timing condition on FRN/RewP amplitudes are illustrated. Error bars and shaded areas represent 95% confidence intervals.

The parsimonious linear mixed-effects model identified for the single-trial FRN/RewP data (see Fig. 2a for the grand-averaged ERPs from the cluster of frontocentral electrodes considered in the analysis and Methods section for details) comparing amplitudes following immediate and delayed feedback involved, as specified, fixed effects of all considered factors (Feedback Valence, Feedback Timing, and PE) and all possible interactions between them. As random effects, the model comprised by-electrode and by-participant random intercepts as well as by-participant random slopes for Feedback Valence, Feedback Timing, PE, the interaction between Feedback Timing and Valence, and the interaction between Feedback Timing and PE. In the *lme4* notation, this is specified as:

$$\begin{aligned}{\text{FRN}}/\mathrm{RewP \, Mean \, Amplitude }&\sim \mathrm{ Feedback \, Valence}*\mathrm{Feedback \,Timing}*{\text{PE}}\\ &\quad+\left(\mathrm{Feedback \, Valence}*\mathrm{Feedback \, Timing}+{\text{PE}}+\mathrm{PE }:\mathrm{Feedback \, Timing}|{\text{Participant}}\right)+(1|{\text{Electrode}})\end{aligned}$$

Importantly, the PE is not confounded with Valence because the absolute (unsigned) PE was used in all analyses which represents surprise, independent of valence of feedback (see Methods for more details). An effect of a reward PE (better-than-expected vs worse-than-expected), as opposed to mere surprise, would thus be demonstrated by an interaction between the PE and Feedback Valence. The model revealed significant main effects of all factors. First, there was a significant effect of Valence,* b* = − 0.38, *t*(19.24) = − 2.75, *p* = 0.013, with more positive amplitudes for positive compared to negative feedback. Moreover, the model revealed a significant effect of Feedback Timing, *b* = − 1.01, *t*(19.06) =  − 5.01, *p* < 0.001, such that immediate feedback elicited more positive FRN/RewP amplitudes than delayed feedback. Furthermore, a significant effect of the PE was found,* b* = 0.94, *t*(16.00) = 3.40, *p* = 0.004, such that the more unexpected the feedback was the more positive were FRN/RewP amplitudes. In addition to that, there was a significant interaction between Feedback Timing and Feedback Valence, *b* = 0.20, *t*(19.69) = 2.54, *p* = 0.019, and between Feedback Timing and the PE, *b* = − 0.57, *t*(18.125) = − 2.13, *p* = 0.047. Follow-up simple slope analyses separately for the two feedback timings showed a significant amplitude difference between positive and negative feedback for immediate (*b* = 1.03, *z* = 2.61, *p* = 0.018) but not for delayed feedback (*b* = 0.19, *z* = 0.85, *p* = 0.789; see Fig. 2b for an illustration of the Feedback Valence by Feedback Timing interaction). With regard to the interaction between Feedback Timing and the PE, follow-up simple slope analyses showed a significant effect of the PE on FRN/RewP amplitudes only for immediate (*b* = 1.51, *z* = 3.54, *p* < 0.001) but not for delayed feedback (*b* = 0.34, *z* = 1.09, *p* = 0.548; see Fig. 2c for an illustration of the PE by Feedback Timing interaction), indicating a general effect of surprise/expectedness only for immediate but not delayed feedback.

Importantly, a significant interaction between Feedback Valence and the PE was found,* b* = − 0.77, *t*(46,479.26) = − 7.21, *p* < 0.001. Follow-up simple slope analyses indicated a significant effect of the PE only for positive (*b* = 1.71, *z* = 5.65, *p* < 0.001), but not negative feedback (*b* = 0.17, *z* = 0.58, *p* = 1.000), suggesting that the violation of outcome expectation only moderates amplitudes following rewards but not punishments with more positive amplitudes for more unexpected rewards. As can be seen in Fig. 2d, this resulted in considerably more positive FRN/RewP amplitudes for better-than-expected compared to worse-than-expected feedback.

The three-way interaction between Valence, the PE, and Feedback Timing was not significant (*b* = − 0.07, *t*(46,872.51) = − 0.63, *p* = 0.530), providing no evidence that the association between Valence and the PE differs between feedback timings. However, as PE processing with immediate and delayed feedback was the main focus of the present study, we were interested to explore whether the same interaction between Feedback Valence and the PE can be found for both the immediate and delayed feedback timing conditions separately. To do so we conducted exploratory follow-up analyses on the interaction between Feedback Valence and the PE separately for both feedback timings. As illustrated in Fig. 2e, these exploratory analyses indicated for immediate as well as delayed feedback a significant interaction between Valence and the PE (for immediate feedback: *b* = − 0.70,* t*(44,313.11) = − 4.41, *p* < 0.001; for delayed feedback: *b* = − 0.84,* t*(37,567.41) = − 5.88, *p* < 0.001). Resolving these interactions revealed the same pattern separately for immediate and delayed feedback as seen in the interaction between Valence and the PE across feedback timings, i.e. a significant modulation of FRN/RewP amplitudes by the PE only for positive but not for negative feedback (slope of the PE for immediate positive feedback: *b* = 2.21, *z* = 4.75, *p* < 0.001; immediate negative feedback *b* = 0.81, *z* = 1.81, *p* = 0.280; delayed positive feedback: *b* = 1.21, *z* = 3.23, *p* = 0.005 ; delayed negative feedback *b* = − 0.47, *z* = − 1.31, *p* = 0.766), resulting in more positive amplitudes for better-than-expected compared to worse-than-expected feedback.

### P300 and model-derived trial-level PE

**Figure 3:**
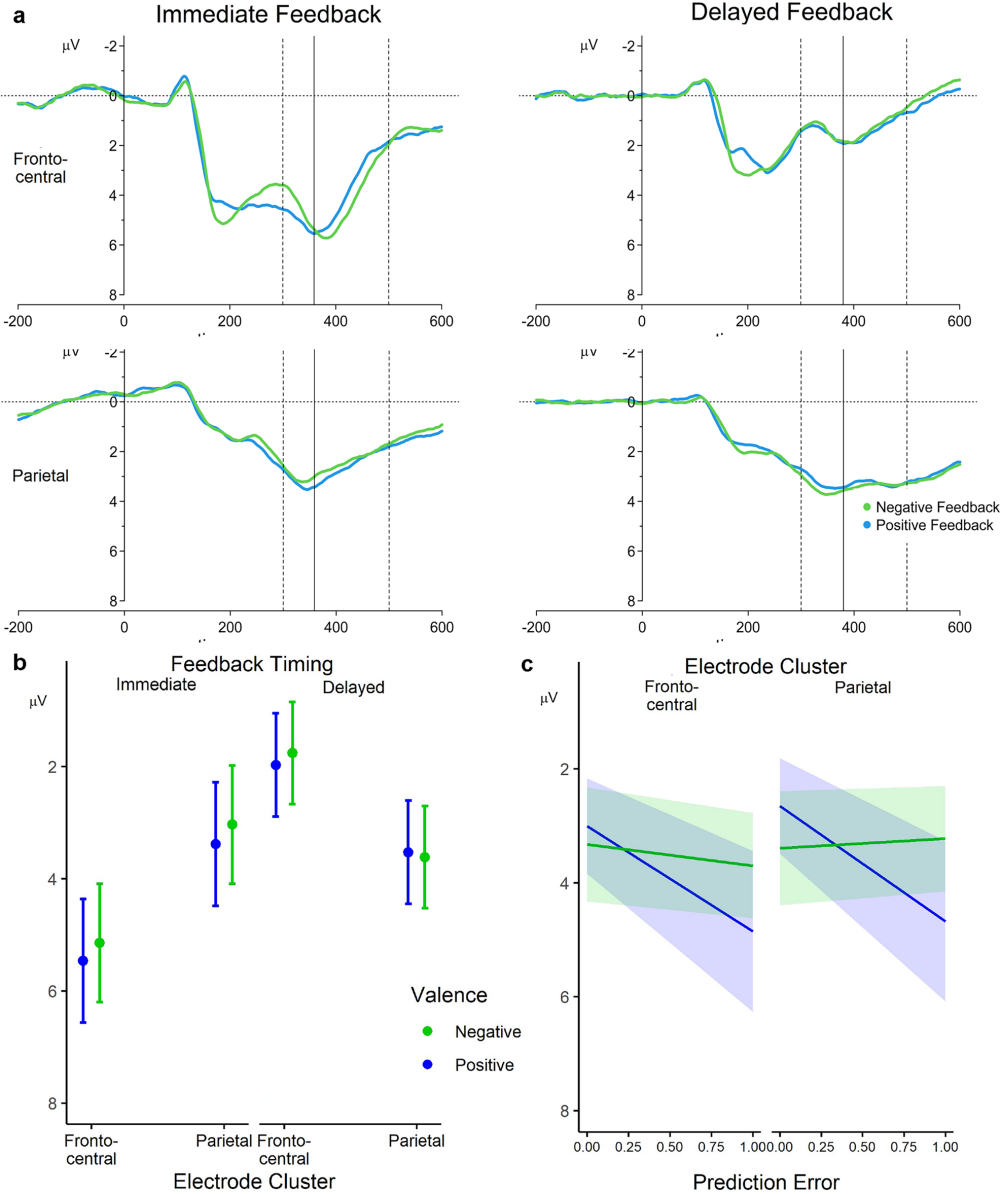
P300 results. (**a**) Feedback-locked grand-averaged ERPs separately for immediate and delayed positive and negative feedback at the frontocentral and parietal electrode cluster. Dashed lines indicate the search time window for quantification. The peak P300 latency, which was the basis for determining the time window for single-trial-amplitude extraction, is shown with the solid line. Note that the peak latency was determined based on the signal average across negative and positive feedback and pooled across the electrodes of both electrode clusters (see Figure S1c in the Supplementary Materials). (**b**) Model-estimated marginal effects illustrating the interaction between the fixed effects of Feedback Valence, Feedback Timing, and Frontality, and **c** between Feedback Valence, PE, and Frontality. Error bars and shaded areas represent 95% confidence intervals.

For the analysis of the P300, ERPs from two clusters of electrode sites were considered (frontocentral and parietal, see Methods section for details). The parsimonious model identified for the single-trial P300 data (see Fig. 3a for the grand-averaged ERPs) involved, as specified, fixed main effects of all factors (Feedback Valence, Feedback Timing, Frontality, and PE) as well as all possible interactions between them. As random effects, the model included by-electrode and by-participant intercepts as well as by-participant slopes for Feedback Valence, Feedback Timing and the PE and all interactions between them. In the *lme4* notation, this is specified as:

$$\begin{aligned}{\text{P}}300 \, \mathrm{ Mean \,Amplitude }&\sim \mathrm{ Feedback \, Valence}*\mathrm{Feedback \, Timing}*{\text{Frontality}}*{\text{PE}}\\ &\quad+\left(\mathrm{Feedback \, Valence}*\mathrm{Feedback \, Timing}*{\text{PE}}|{\text{Participant}}\right)+(1|{\text{Electrode}})\end{aligned}$$

The model revealed significant main effects of Feedback Timing, *b* = − 0.80,* t*(18.98) = − 5.01, *p* < 0.001, and Feedback Valence, *b* = − 0.19,* t*(18.93) = − 2.64, *p* = 0.016, which were further moderated by an interaction between Feedback Timing and Frontality,* b* = − 0.96,* t*(116,838.05) = − 47.47, *p* < 0.001, and between these factors and Feedback Valence,* b* = − 0.06,* t*(116,838.05) = − 2.75, *p* = 0.006. As can also be seen in Fig. 3b, for immediate feedback the P300 was more pronounced (i.e. more positive) at frontocentral than parietal electrodes (*b* = − 2.09, *z* = − 6.01, *p* < 0.001), while this pattern was reversed for delayed feedback (*b* = 1.71, *z* = 4.90, *p* < 0.001), as revealed by follow-up simple slope analyses to resolve the two-way interaction. Follow-up simple slope analyses to resolve the three-way interaction confirmed that the reversed pattern, i.e. the difference in amplitudes between the frontocentral and parietal cluster in delayed feedback, was slightly stronger for negative compared to positive feedback (effect of Frontality (1) for immediate positive feedback:* b* = − 2.08, *z* = − 5.89, *p* < 0.001, (2) for immediate negative feedback:* b* = − 2.11, *z* = − 5.99, *p* < 0.001, (3) reversed effect for delayed positive feedback: *b* = 1.56, *z* = 4.41, *p* < 0.001, (4) for delayed negative feedback: *b* = 1.86, *z* = 5.28, *p* < 0.001).

Furthermore, the model revealed a significant effect of the PE on P300 amplitudes,* b* = 1.02, *t*(9.33) = 3.50, *p* = 0.006, indicating larger P300 amplitudes the larger the PE is (as can be seen in Fig. 3c). In addition, the model revealed significant interactions between the PE and Feedback Valence, *b* = − 0.92, *t*(12.64) = − 2.73, *p* = 0.017, and between these factors and Frontality, *b* = 0.18,* t*(116,838.05) = 2.51, *p* = 0.012 (see Fig. 3c). Follow-up simple slope analyses showed a significant PE effect on P300 amplitudes only for positive (*b* = 1.93, *z* = 3.37, *p* = 0.001) but not for negative feedback (*b* = 0.10, *z* = 0.39, *p* = 1.000), explaining the two-way interaction. This pattern was consistent for amplitudes from the frontocentral and parietal electrode cluster (effect of PE (1) for positive feedback at frontocentral electrodes:* b* = 1.85, *z* = 3.17, *p* = 0.006, (2) for negative feedback at frontocentral electrodes:* b* = 0.37, *z* = 1.35, *p* = 0.707, (3) for positive feedback at parietal electrodes: *b* = 2.02, *z* = 3.46, *p* = 0.002, (4) for negative feedback at parietal electrodes: *b* = − 0.17, *z* = − 0.62, *p* = 1.000). As can be seen in Fig. 3c, the relationship between the PE and P300 amplitudes is positive (i.e. larger PEs are associated with more positive amplitudes) for positive and negative feedback at the frontocentral cluster and for positive feedback at the parietal cluster. Only for P300 amplitudes in response to negative feedback and only at parietal sites, the estimated effect exhibited a different sign, and thus, a reversed relationship. While the effect of the PE for negative feedback at parietal sites was not significantly different from zero (see above), post-hoc pairwise comparisons revealed that this slope differed from all others (i.e. slope of PE for negative feedback at parietal electrodes (1) vs slope of PE for positive feedback at parietal electrode: *b* = 2.19, *z* = 3.19, *p* = 0.009, (2) vs slope of PE for negative feedback at frontocentral electrodes: *b* = − 0.54, *z* = − 2.93, *p* = 0.020, and (3) vs slope of PE for positive feedback at frontocentral electrodes: *b* = − 2.02, *z* = − 2.94, *p* = 0.020), which has likely driven the three-way interaction of the PE and Valence pattern with the factor Frontality.

## Discussion

In this study we investigated PE representations in the neural processing of immediate and delayed feedback during a reinforcement learning task. In a previous study, using the same reinforcement learning task^31^, we had shown that the difference wave for the processing of negative and positive feedback in the time window of the FRN/RewP is modulated by stimulus reward probabilities as a proxy for feedback expectancy for both immediate and delayed feedback, although the difference wave amplitude as such was reduced for delayed feedback. In the present work, we used data previously published^32^ and first applied computational models to the behavioural choice data to derive PE values for each individual trial and then related these to single-trial ERP amplitudes for each individual participant. The question of main interest was in how far the PE is reflected in the FRN/RewP time window for the processing of immediate and delayed feedback. In an additional analysis, a later ERP component was also analysed, the P300 which has frequently been linked to reward related processes (see e.g., refs.^40,45, 46^).

### Feedback timing effects and PE representations in the FRN/RewP time window

In our single-trial analysis of the FRN/RewP we could first of all replicate effects well known from the literature. The amplitude was more positive for positive feedback^5,23^ and this valence-dependent amplitude difference was more pronounced for immediate than delayed feedback^2,26, 31–35^. Moreover, our analysis revealed an interaction between the PE and Feedback Timing, indicating a general effect of surprise for immediate feedback, which was not seen for delayed feedback. Of particular interest was that we found an effect of the unsigned PE in interaction with feedback valence. For positive feedback the amplitude was more positive the larger the PE, that is, the more unexpected the outcome was. In contrast, for negative feedback no effect of the PE was found, that is, amplitudes did not scale with the PE. As a result, amplitudes in response to unexpected rewards were more positive compared to unexpected losses. This result pattern is consistent with a study reporting effects of valence and expectancy on the signal in the FRN/RewP time window in a large sample of nearly 1000 participants performing a time estimation task^40^. Similar to our finding across feedback timing conditions they reported a stronger expectancy effect for positive than negative feedback trials, with more positive amplitudes for more unexpected feedback.

Importantly, the modulation of FRN/RewP amplitudes in response to rewards by the model-derived single-trial PEs was not affected by whether the feedback was given immediately or delayed. As the absence of a significant interaction between the PE, valence and feedback timing does not necessarily indicate that the PE effects were comparable for immediate and delayed positive feedback, we additionally conducted exploratory follow-up analyses for the two-way interaction and the simple slopes of the PE for positive and negative feedback for the two feedback timing conditions separately. These analyses revealed remarkably similar patterns of results concerning the interaction between feedback valence and the PE. As for the overall pattern across both conditions, significant PE effects were only seen for positive feedback in both immediate and delayed feedback processing, while no effect of the PE emerged for negative feedback. We could thus replicate previous findings concerning differences in feedback processing between immediate and delayed feedback concerning the strength of the valence effect (see above) but showed for the first time that single-trial PEs are similarly reflected in immediate and delayed processing of rewards. This pattern of results is also consistent with previous findings of our lab, where we found effects of expectancy (operationalized as a binary variable based on low vs high stimulus reward probabilities across experimental trials) in immediate as well as delayed feedback processing in a different sample and with a between-subjects design^31^. Given that the FRN/RewP has been suggested to reflect striatal processing^28,30^, this finding indicates that the striatum is similarly involved in immediate and delayed feedback processing. This appears to speak against the notion that the striatum underlies learning from immediate and the hippocampus learning from delayed feedback^10^, at least at first sight. However, also previous functional imaging studies suggested PE coding in the striatum across feedback timing conditions^11,13^, and at least in one of them the stronger PE representation for immediate feedback was restricted to the dorsal striatum^11^, while the FRN/RewP correlates with reward-related activity in the ventral striatum^28^. It is thus conceivable that at least part of the striatum is similarly important for learning from immediate and delayed feedback. Additionally, our previous findings obtained in PD patients support this view. We found that the bias of enhanced learning from negative feedback in unmedicated PD patients^37^, which has been ascribed to DA depletion in the striatum, can also be seen for learning from delayed feedback^38^. Overall, these findings may mean that different neural mechanisms underlying feedback learning are represented in the striatum, with only one of them being affected by feedback timing. Alternatively, the striatum may be involved in learning from delayed feedback, but to a lesser extent than in learning from immediate feedback.

Irrespective of feedback timing, the results of this study provide strong evidence in favour of the notion that the signal in the time window between 200 and 350 ms after feedback presentation at frontocentral electrode sites can best be described as RewP. As outlined above, PE-dependent modulations of the signal were only seen for positive feedback, in line with the study by Kirsch et al.^40^. This may indicate that the ERP in response to negative feedback can rather be regarded as a baseline, and the negativity that has in some previous studies been termed FRN_peak_^26,33^ is in fact an N200, as suggested by Proudfit^17^. The focus on rewards, or in other words, on the confirmation of an existing expectation, has been described as a robust feature of human reinforcement learning^44,47^. This choice-confirmation bias was also reflected in participants’ learning rates. For both feedback timing conditions, reward expectations were updated significantly more strongly following positive PEs (better-than-expected) than after negative PEs (worse-than-expected). Thus, we process and learn preferentially from feedback confirming our prior beliefs, regardless of whether we receive this confirmation immediately or delayed.

### Feedback-related processes reflected by the P300

For the P300, Feedback Timing effects were found in interaction with Frontality and Feedback Valence. As suggested by visual inspection (see Fig. 3b), there was an amplitude difference between immediate and delayed feedback at frontocentral electrodes while the amplitudes for the feedback timing conditions were comparable at parietal electrodes. Moreover, amplitudes were slightly larger for positive than negative feedback, and only for delayed feedback at parietal electrode sites the opposite pattern emerged. Given that the frontal and parietal P300, referred to as P3a and P3b, have been linked to different cognitive processes in the evaluation of feedback, this result indicates that immediate and delayed feedback were processed differently in this later time window. The frontal P3a has been suggested to reflect attention allocation to stimuli^48^. The enhanced P300 at frontal sites for immediate feedback thus appears to suggest that immediate feedback elicited a stronger orienting response than delayed feedback.

The centroparietal P3b, in turn, seems to reflect stimulus value updating and subsequent behavioural adaptation in reinforcement learning tasks^25,40, 49^. Compared to the earlier processes in the FRN/RewP time-window, the P3b may reflect a more declarative process of value updating in the context of model-based learning^50^, possibly based on PEs^43^. However, despite a significant interaction between the PE, Valence and Frontality in our analysis, the effect of the PE was comparable over the parietal and the frontocentral cortex. More specifically, the effect that we observed in the P300 time range was very similar to the one observed for the FRN/RewP. The amplitude was larger for more unexpected feedback, but only for positive feedback, indicating an encoding of a reward PE also for this later time range. The three-way interaction with Frontality was driven by a sign change in the effect estimate of the PE only for negative feedback at parietal sites (i.e. predicting more negative amplitudes for more unexpected feedback), which, however, was not significant per se. Previous studies finding a PE effect in the P300 time range mainly found such an effect at parietal sites, i.e. for the P3b. However, it should be noted that PE effects on the P300 were not found as consistently and of lower magnitude as for the FRN/RewP, with several studies reporting that the P300 amplitude was mainly driven by feedback valence^24,25^.

There are several reasons that might contribute to these inconsistencies. First, the problem of temporal overlap of components, common in ERP analyses, makes the assignment of PE effects to a specific component within this time range ambiguous, especially given that the time windows of the subcomponents of the P300 additionally overlap with the time window in which the FRN/RewP is typically quantified. Additionally, differences in learning paradigms, such as different learning and feedback stimuli, task difficulty or rule changes, might produce a different set of overlapping components and potentially latency shifts. Such differences might explain why some studies find PE effects rather at parietal sites in a time window associated with the P3^51^, while others find PE modulations only in the FRN time window^24^. To date, it is unclear whether this signature of the PE in the P300, especially at frontal electrodes, constitutes a separate or a sustained process from the FRN/RewP time range^43^.

Lastly, there is also heterogeneity in previous studies regarding how the PE is computed. While a growing body of research uses computational modelling to derive PEs, that considers individual learning processes to infer latent reward expectations of participants in a trial-by-trial fashion^51^, other studies rely solely on statistical reward probabilities of stimuli inherent in the experimental design, serving as a proxy for expectedness/PEs. As we demonstrate with an additional exploratory analysis (Supplementary Material S2) in which we replaced our model-derived PEs with the fixed reward probabilities, the two operationalizations map different processes. While we find no association between fixed reward probabilities and FRN/RewP amplitudes, effects on the P300 are seen mainly for parietal but not frontocentral electrodes. As the fixed reward probabilities do not reflect learning-related changes in implicit reward expectations, this strengthens the notion that the parietal P3b might reflect rather declarative learning updates while the FRN/RewP and the frontocentral P3a might capture more implicit expectation (violation). Differences between previous studies in how PEs are derived therefore likely contribute to the divergent findings regarding the PE and the P300.

As outlined above, the frontal (P3a) and parietal (P3b) P300 may represent different processes in the context of the evaluation of feedback and how it is used for learning, which are possibly differentially involved in immediate and delayed feedback processing. It is thus remarkable that we found PE processing, at least for positive feedback, for both feedback timings. In how far the processing for delayed feedback is more declarative in nature, and by which brain regions it is modulated, needs to be addressed in future research.

### General aspects and limitations

With an analysis approach based on the computation of trial-by-trial subjective stimulus values and PEs to relate them to single-trial ERP amplitudes within linear mixed-effects models this study yielded new insights into similarities and differences in feedback processing between immediate and delayed feedback. The main finding is that for immediate and delayed feedback alike the ERP signal in the time window of the FRN/RewP reflects a PE, but only for positive feedback. For the P300 a similar pattern was found, but also differences between immediate and delayed feedback processing were seen. This raises the question how negative PEs are processed, as we did not find PE effects on negative feedback processing in any of the analysed ERP components^52^. Initially, the FRN was considered to reflect an error signal mainly reflecting negative feedback processing^20^. In the last years the term RewP is more and more used for the ERP signal between about 200 and 350 ms after feedback onset, as accumulating evidence suggests that this signal primarily reflects processes related to positive feedback^17,28^. Another suggestion supported by a recent study also applying single-trial analysis states that two processes, one related to positive and one to negative feedback processing, overlap in the mentioned time window^27^. Also for the P300 the findings are mixed, as some studies report that it is more strongly related to positive feedback^1,21, 40, 53^ while others relate it primarily to negative feedback processing^24^. We suggest that this distinct processing pattern for positive feedback likely reflects the human bias to learn preferentially from outcomes that confirm our previous beliefs^44,47^ as this was also reflected in significantly larger learning rates for positive feedback while those for negative feedback were negligible.

By using computational models to derive latent reward expectations for each participant in each individual trial based on their actual choice behaviour, we could simulate individual learning processes and observe that these differ between positive and negative feedback trials. However, the estimated stimulus values converged, as expected, to the fixed reward probability values on average after about one third of the trials (as can be seen in Fig. 1e). One might therefore wonder to what extent the PEs derived with the computational modelling approach provide insights beyond the above mentioned differences in learning processes, in contrast to using the task-inherent reward probabilities. As demonstrated in an exploratory analysis (Supplementary Material S2), the objective reward probabilities do not map onto the neural signals in the FRN/RewP time window as the model-derived PEs do. For the P300, we found partially similar results as for the model-derived PEs (i.e. an effect of Reward Probability for positive but not negative feedback), however, additionally a general effect of Reward Probability regardless of valence only at parietal but not frontocentral electrode sites. Thus, the model-derived PEs convey learning-related information that the fixed reward probabilities neglect, especially for the phase in which most knowledge is gained (i.e. the first block). Moreover, by assuming equal reward expectations for the beginning of the experiment, the confounded relationship between (subjective) probabilities and valence inherent in the task design (e.g. 80% stimulus is paired with positive feedback more often than the 20% stimulus) is attenuated for the estimated stimulus values which converge to the objective reward probabilities only after a substantial amount of learning trials.

While the pattern observed in the present data regarding effects of the PE on positive feedback processing is in line with previous studies examining the processing of immediate feedback^40^, the consistency of this pattern for delayed feedback demands replication. While we included a relatively large number of trials per participant, the number of participants was relatively small (n = 20). Statistical power in linear mixed-models, however, is more strongly dependent on second level units^54^, i.e. here: participants. Thus, especially for the non-significant three-way interaction between Feedback Timing, PE and Valence, we cannot exclude that the statistical power did not suffice to find a possible “true” effect. To explore how likely it would have been to detect a small effect (b = 0.3) of this interaction with our design and sample, we conducted a post-hoc power analysis^55^, which estimated a power of 76.00% (CI: 69.47, 81.74) to have detected such an interaction. Notwithstanding, the small number of participants poses a limitation and the conclusions about our non-significant findings have to be treated with caution.

In conclusion, using computational modelling and single-trial EEG analysis, we present novel evidence of PE representations in immediate and delayed feedback processing. For the time window of the FRN/RewP our findings suggest that, despite a reduced effect of feedback valence for delayed feedback, there are strong similarities between immediate and delayed feedback concerning PE processing. For both feedback timings, positive PEs are reflected in the ERP, i.e. more unexpected rewards are associated with more positive amplitudes. A similar pattern is found in the later time window of the P300. The P300 was generally more pronounced over the frontal than the parietal cortex for immediate feedback, while this pattern was not seen for delayed feedback. However, PE representations were present regardless of Feedback Timing and of comparable magnitude over the frontal and the parietal cortex for rewards but not losses. Overall, our results are in line with the concept of the RewP that primarily drives feedback processing between 200 and 350 ms after feedback onset, irrespective of feedback timing and the human bias to preferentially process and learn from feedback reinforcing our choices.

## Method

### Participants

Data from twenty participants (*M*_age_ = 24.8, *SD* = 2.7; 11 female, 9 male) with normal or corrected-to-normal vision and no history of neurological or psychiatric disorders or regular consumption of alcohol or psychodynamic drugs were considered in this study. In the present manuscript we present a reanalysis of part of the data reported in the study by Weismüller et al.^32^ on the neural correlates of feedback-based probabilistic learning and the effect of feedback timing. While the original study comprised 40 participants, half of them learning by observation, the focus in the reanalysis is on single-trial ERPs and their relationship to PEs derived from computational models in the 20 participants learning actively from their choices. Participants gave informed written consent prior to their participation and received course credit or money (15 €) as compensation. The study conformed to the guidelines in the Declaration of Helsinki and has been approved by the ethics committee of the faculty of mathematics and natural sciences at Heinrich Heine University Düsseldorf.

### Probabilistic learning task

In the previously described probabilistic learning task^32^ participants were asked to choose one from two visual stimuli presented on the left and right side of a computer screen in each trial. As visual stimuli we used symbols representing or resembling Japanese Hiragana signs. For their choice participants received positive or negative feedback in the form of monetary reward (+ 20¢) or punishment (− 10¢) after 1 (immediate) versus 7 s (delayed feedback). Participants underwent 300 of such learning trials per feedback timing condition. Trials were thereby organized in three blocks (with 100 trials each) which each ensued a test phase without trial-by-trial feedback. The latter was included in the original study to compare learning performance between active and observational learners and is not analyzed here, as only the data of the active learners are of interest in the present analysis. Participants completed all learning trials and test phases of one feedback timing condition before undergoing the other condition. The order of timing conditions was counterbalanced between participants. For each timing condition, there was a separate set of five stimuli with different reward probabilities (i.e. 0%, 20%, 40%, 60%, and 80% reward probability). That is, for instance, the choice of the stimulus with a reward probability of 20% was followed by positive feedback (reward) in 20% of the trials in which it was chosen and by negative feedback (punishment) in 80% of the trials in which it was chosen. Each of the ten possible combinations of stimuli was presented equally often in each learning phase (i.e. ten times), with counterbalanced positions of the stimuli in each pair with respect to the side on the screen. While the exact probabilities remained unknown to the participants, they were able to learn which stimuli were preferable and which not, based on the feedback they received. Figure 1a shows one of the sets of stimuli used as well as a schematic overview of a learning trial (note that there was a separate set for the other feedback timing condition, for further details see ref.^32^).

### Computational models to determine trial-by-trial PEs

To derive trial-by-trial values of reward PEs for each participant in each feedback timing condition, reinforcement learning models^56^ were fitted to the behavioural data (i.e. the participants’ sequence of choices) and the given feedback using MATLAB R2018b (MathWorks Inc., Natick, USA). Three models with increasing complexity were compared, aiming to obtain PE estimates of a model whose predicted choices deviate the least from the observed behaviour (for similar approaches, see e.g.^24,57^).

In the first model (M_1_), each of the five stimuli is assigned a stimulus value, ${Q}_{1,\dots ,5}$, that is iteratively updated in every trial $t$ in which the respective stimulus was chosen. Initial $Q$ values were set to 0.5 for all stimuli. The update of the stimulus value of the chosen stimulus, ${Q}_{c}$, was then based on the deviation between the prior value and the received outcome, i.e. the PE $\delta$, and a constant learning rate $\alpha$, reflecting the degree to which the PE was used to update the stimulus value:

1 $${Q}_{c,t+1}={Q}_{c,t}+ \alpha * {\delta }_{c,t},$$

with the PE ${\delta }_{c,t}$ being calculated as.

2 $${\delta }_{c,t}={r}_{t}-{Q}_{c,t},$$

where the reward ${r}_{t}$ is 1 for positive feedback in the given trial $t$ and 0 for negative feedback.

For each trial ${t}_{1,\dots ,{n}_{trials}}$, the probability $p$ that the model would choose the stimulus that was observed to be chosen (i.e., that a participant actually has chosen) was calculated using the softmax function based on prior stimulus values of both stimuli that were available to choose, that is, values of the chosen stimulus, ${Q}_{c,t}$, and the unchosen stimulus in trial *t*, ${Q}_{u,t}$, and an exploration parameter $\beta$:

3 $${p}_{c,t}= \frac{{e}^{{{\varvec{Q}}}_{{\varvec{c}},{\varvec{t}}}*\beta }}{{e}^{{{\varvec{Q}}}_{{\varvec{c}},{\varvec{t}}}*\beta }+{e}^{{{\varvec{Q}}}_{{\varvec{u}},{\varvec{t}}}*\beta }}$$

The size of $\beta$ thereby reflects the impact of prior stimulus values on a subject’s choices, that is, whether a participant either exploited prior stimulus values (resulting in a larger $\beta$, and, thus, a larger impact of prior values) or whether a participant showed rather explorative behaviour (with a smaller $\beta$, and, thus, a smaller impact of prior values on their choices). These probabilities were then used to calculate the negative summed log-likelihood ($-LL$) indicating the model’s goodness of fit:

4 $$-\sum log({p}_{c,{t}_{1,\dots ,{n}_{trials}}})$$

The optimization function *fmincon* from the Optimization Toolbox of MATLAB was used to minimize the $-LL$ value for all tested models, that is, to estimate values for the free parameters (i.e. for M_1_: $\alpha ,$ $\beta$) that result in least deviation between the model’s predicted choices and the observed behaviour. To avoid local minima, each model was fitted repeatedly to the subjects’ behaviour (50 iterations) with random numbers in the interval $\left[0;1\right]$ as start values for the free parameters. Value constraints for the free parameters were set to $\left[0;1\right]$ for the learning rate $\alpha$ and to $\left[0;100\right]$ for the exploration parameter $\beta$.

In the second model (M_2_), the learning rate for positive feedback (reward) and negative feedback (punishment) was allowed to differ, accounting for a potential choice-confirmation bias^44^ to learn preferentially from positive PEs (i.e. feedback that is better than expected and confirms the choice) compared to negative PEs (i.e. feedback that is worse than expected and disconfirms the choice)^57,58^. For trials with positive feedback, the stimulus value of the chosen stimulus was therefore updated with the learning rate ${\alpha }_{con}$ as follows:

5 $${Q}_{c,t+1}={Q}_{c,t}+ {\alpha }_{con} * {\delta }_{c,t}$$

And analogously, for trials with negative feedback, the stimulus value of the chosen stimulus was updated with the learning rate ${\alpha }_{dis}$:

6 $${Q}_{c,t+1}={Q}_{c,t}+ {\alpha }_{dis} * {\delta }_{c,t}$$

For both learning rates, boundary constraints were set to $\left[0;1\right]$ as for the global learning rate in M_1_. Everything else remained unchanged.

In the third model (M_3_), stimulus values of both stimuli available to choose in a given trial were updated, that is, the value of the chosen stimulus, ${Q}_{c,t}$, as well as of the unchosen stimulus, ${Q}_{u,t}$. Since participants were instructed that the feedback they receive in a given trial reflects whether their choice between the two presented stimuli was correct, it can be assumed that inferences from the feedback for the chosen stimulus were drawn also for the unchosen stimulus. In other words, positive feedback for the chosen stimulus can be regarded as confirmation of both the choice of the chosen stimulus and the non-choice of the unchosen stimulus (and vice versa for negative feedback). The update of the stimulus value for the unchosen stimulus, ${Q}_{u,t}$, was therefore calculated for trials with positive feedback (i.e. reward for the chosen stimulus) as follows:

7 $${Q}_{u,t+1}={Q}_{u,t}+ {\alpha }_{con} *{\delta }_{u,t}$$

The update in trials with punishment for the chosen stimulus was done analogously with ${\alpha }_{dis}$. The PE for the unchosen stimulus (cf. ref.^24^) was thereby computed as:

8 $${\delta }_{u,t} =1-{r}_{t}-{Q}_{u,t}$$

Everything else remained unchanged compared to M_2_.

The three models were compared based on their negative summed log-likelihood ($-LL$), calculated as described above, as well as the Bayesian information criterion (BIC) which, in contrast to the $-LL$, accounts for the number of free parameters to avoid overfitting. For both criteria, lower values indicate a better fit of the model to the observed data. The model with the lowest $-LL$ and BIC values was M_3_ (see Table 1) which was therefore used to extract stimulus values which are visualized in Fig. 1e as well as trial-by-trial PEs. For statistical analysis, absolute values of PEs were used (i.e., unsigned PEs) for two reasons. First, the signed PE and feedback valence are confounded, and effects of the signed PE on ERP amplitudes can thus in fact be driven by feedback valence. Second, our procedure allows the influence of expectation violation to be examined separately for positive and negative feedback (see below).

**Table 1:** Model fit to observed choice behavior.

Model	− LL	BIC
M_1_	167.48	340.97
M_2_	158.97	323.95
M_3_	154.12	314.24

### EEG data acquisition and preprocessing

EEG was recorded with 29 Ag/AgCl active electrodes positioned according to the international 10–10 system^59^ at electrode sites F7, F3, Fz, F4, F8, FC5, FC1, FC2, FC6, T7, C3, Cz, C4, T8, CP5, CP1, CP2, CP6, P7, P3, Pz, P4, P8, PO9, O1, Oz, O2, PO10, and FCz (online reference). One electrode placed lateral to the left outer canthus and one electrode placed above the left eye (Fp1) served to measure horizontal and vertical eye movements as well as eye blinks. The signal was amplified with a BrainAmp DC amplifier and recorded via BrainVision Recorder software (Version 1.20, Brain Products GmbH, Germany) with an online low cutoff filter of 0 Hz and an online high cutoff filter of 1,000 Hz. Impedances were kept below 10 kΩ.

Preprocessing of the recorded EEG data was performed with BrainVision Analyzer software (Version 2.2, Brain Products GmbH, Germany) and R (Version 4.0.3, R Core Team, 2020). The data was re-referenced to the average reference, corrected for direct current trends and filtered with zero-phase Butterworth filters (high-pass: 0.1 Hz, order: 2 (12 dB/oct), time constant: 1.59 s; low-pass: 30 Hz, order: 2 (12 dB/oct); notch filter: 50 Hz). The continuous data was then segmented into epochs from 200 ms before to 800 ms after feedback stimulus onset. The first 200 ms of each epoch were then used for a baseline correction. To prepare the data for the subsequent eye movement and blink artifact correction, an automatic artifact rejection was applied to all but frontal and frontocentral electrodes using the following parameters: maximal allowed voltage step: 70 µV/ms; maximal allowed absolute difference within 100 ms intervals: 200 µV; minimal allowed amplitude: -150 µV; maximal allowed amplitude: 150 µV; lowest allowed activity in 100 ms intervals: 0.1 µV. The algorithm by Gratton & Coles^60^ was then applied to epochs of all electrodes to correct for eye movement and blink artifacts followed by the same artifact rejection as described above as well as another baseline correction with the 200 ms pre-stimulus interval (for a similar procedure see ref. ^31^). Data of all remaining epochs of electrodes of interest (see below) was then exported for further processing and analysis in R.

Following the procedure of studies analyzing feedback timing effects on the FRN/RewP (e.g. refs.^31,33, 34^), we quantified FRN/RewP amplitudes based on the punishment-reward difference wave at frontocentral electrodes (see Fig. 2a). More specifically, we included data from electrode sites Fz, FCz, Cz, FC1, and FC2 (for separate grand averages for each electrode, see Supplementary Fig. S1a). Waveforms of trials with immediate and delayed negative and positive feedback were first averaged separately (across participants and electrodes) and then subtracted from each other, separately for each feedback timing condition, yielding two punishment-reward difference waves (see Fig. 2a). In the next step, the largest negative (local) peak of the difference waves was determined in a time window between 180 and 350 ms after feedback stimulus onset for immediate and delayed feedback. The latencies of the two peaks then served to extract FRN/RewP amplitude values from each electrode in each trial of the respective feedback timing condition, which were calculated as mean amplitudes in a time window from 30 ms before to 30 ms after the feedback timing-specific peak latency (273–333 ms for immediate and 242–302 ms for delayed feedback).

To quantify the P300, we averaged waveforms across trials with negative and positive feedback and across two clusters of electrodes, i.e. a frontocentral cluster (Fz, FCz, Cz, FC1, and FC2) and a parietal cluster (CP1, CP2, P3, Pz, and P4), separately for each feedback timing condition (for separate grand averages for each electrode, see Supplementary Fig. S1a and S1b). We then identified peak latencies (local maxima) in these averages between 300 and 500 ms after feedback stimulus onset (see Supplementary Fig. S1c), which were subsequently used to extract P300 mean amplitudes from each electrode in each trial in the respective feedback timing condition in a time window from 30 ms before to 30 ms after the peak latency of the feedback timing-specific averages (350–410 ms for immediate and 329–389 ms for delayed feedback).

### Statistical analyses

All statistical analyses were performed in R. Learning performance was examined with a repeated-measures ANOVA (rANOVA) with the factors Feedback Timing (immediate, delayed) and Block (1–3). The dependent variable was the number of correct responses per learning block. Choices in which the stimulus with the higher reward probability was chosen were regarded as correct. Greenhouse-Geisser correction was applied to the degrees of freedom in case of a violation of sphericity. A binomial test with a probability of success of 0.5 on each trial and an alpha level of 0.05 confirmed that no participant performed significantly below chance in any feedback timing condition. In our published paper on this study we analyzed learning performance already^32^. However, as the focus there was on the comparison between active and observational learners we used data from test trials without feedback, while in the present study we used data from learning trials. In addition, we analysed the non-normally distributed estimated learning rates of the computational models with Wilcoxon signed-rank tests, first separately for Feedback Type (positive vs negative) and Feedback Timing (immediate vs delayed), and subsequently the differences scores between Feedback Type (learning rates for positive minus learning rates for negative feedback) between Feedback Timings (immediate vs delayed). In follow-up analyses p-values were Bonferroni-corrected.

Statistical analyses of ERP data were performed using the packages *lme4*^61^ (version 1.1.26) and *lmerTest*^62^ (version 3.1.3). The package *buildmer*^63^ (version 2.4) was used to select a parsimonious linear mixed-effects model^64^ for the analysis of each ERP component separately. The selection procedure thereby included two steps. First, a maximal model formula (see below) was delivered to *buildmer* and a maximal feasible model that still converged was identified by adding terms (i.e. fixed and random effects) to an empty model in order of their contribution to a significant change in log-likelihood (i.e. with lower chi-square *p* values). In a second step, backward stepwise elimination of random effect terms of this maximal feasible model was performed to identify a parsimonious random effect structure for the given data (again based on significance of log-likelihood change^63^). Degrees of freedom and p-values were derived from Satterthwaite approximations.

The delivered maximal model included fixed effects of the factors Feedback Valence (positive vs negative), Feedback Timing (immediate vs delayed), PE (continuous), and all possible interactions between these, as well as a random intercept for Electrode and Participant and random by-participant slopes for the effects of Feedback Valence, Feedback Timing, and PE (and again with all possible interactions between these). In the notation of the *lme4* package, this is specified as follows:

9 $$\mathrm{ERP\, Amplitude }\sim \mathrm{ Feedback \, Valence}*\mathrm{Feedback \, Timing}*{\text{PE}}+ \left(\mathrm{Feedback \,Valence}*\mathrm{Feedback \,Timing}*{\text{PE}}|{\text{Participant}}\right)+(1|{\text{Electrode}})$$

Importantly, the absolute PE, with higher values indicating larger expectation violations regardless of Feedback Valence, was entered as factor into the analysis, as the signed PE as determined via computational modelling, would be correlated with Feedback Valence. As outlined above, we used valence and the unsigned PE as separate factors. If the signed PE is represented in an ERP amplitude this would be statistically reflected in an interaction between PE and Feedback Valence. However, depending on the pattern that emerges in the resolution of this interaction, the ERP does not necessarily represent the full range of the signed PE^65^. Instead, separate analyses on PE effects for positive and negative feedback can then reveal, whether a particular ERP component reflects the unsigned PE (or surprise) more strongly for one of the two feedback types. For the analysis of the P300, a fixed effect for the factor Frontality and its interactions with all other factors was added.

All predictor variables were centered around zero: The categorical variables (i.e. Feedback Valence, Feedback Timing, Frontality), with two levels each, were effect-coded (i.e. -1 vs 1), whereas the continuous variable PE was shifted around zero while maintaining its original range. Significant interactions were resolved with simple slope analyses for each level of the categorical factors. Significance was indicated by an α level of below 0.05. For follow-up simple slope analyses to resolve interactions a Bonferroni correction was applied. Analysis code including output can be found at https://github.com/coweb101/fbdelaype.

### Ethical approval

The study conformed to the guidelines in the Declaration of Helsinki and has been approved by the ethics committee of the faculty of mathematics and natural sciences at Heinrich Heine University Düsseldorf.

### Supplementary Information

Supplementary Information.

## Data Availability

The data cannot be made publicly available because the consent forms signed by the participants do not cover public availability and permanent archiving of the data, even if fully anonymized. Materials (i.e. full sets of visual stimuli and Presentation code) are available on request from the corresponding author (constanze.weber@hhu.de).

## Code availability

Analysis code including model output is available at https://github.com/coweb101/fbdelaype.
