# Supplementary material for: Prediction-error-dependent processing of immediate and delayed positive feedback
Source: Sci Rep. 2024 Apr 27;14:9674. doi: 10.1038/s41598-024-60328-8 (PMC11055855; doi:10.1038/s41598-024-60328-8)
Supplement: Supplementary file 1 — Supplementary Information. [file 41598_2024_60328_MOESM1_ESM.pdf]

Supplementary online material to:

Prediction-error-dependent processing of immediate and delayed positive feedback

Constanze Weber[a*], Christian Bellebaum[a]

[a]Institute of Experimental Psychology, Department of Biological Psychology, Heinrich Heine University, Düsseldorf, Germany

[*]*Corresponding author*: Constanze Weber, Heinrich Heine University, Institute of Experimental Psychology, Department of Biological Psychology, Universitätstrasse, 1, 40255, Düsseldorf, Germany.

Phone: +49 211 81-11594

Email: Constanze.Weber@hhu.de

S1. Grand-averaged ERPs

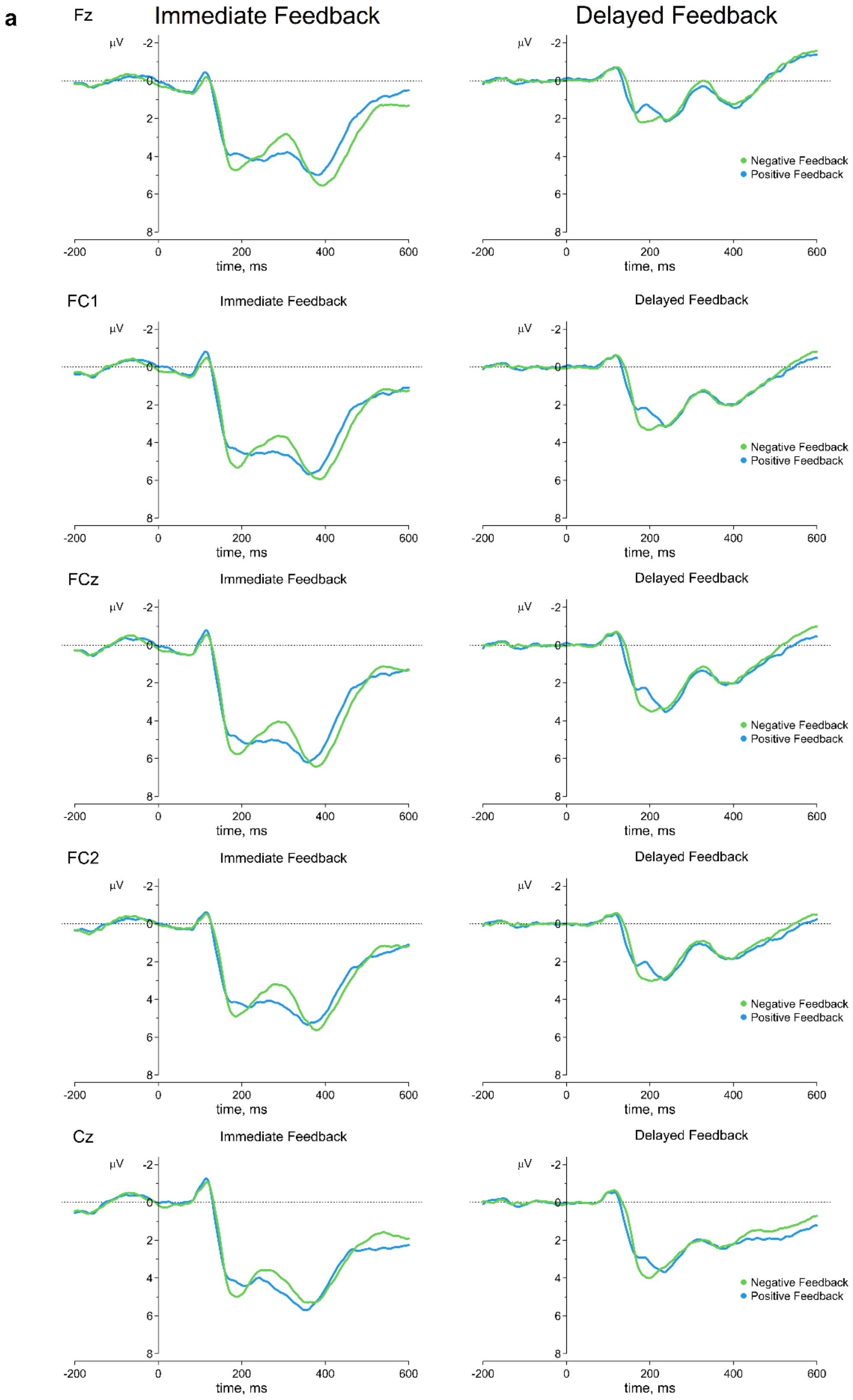

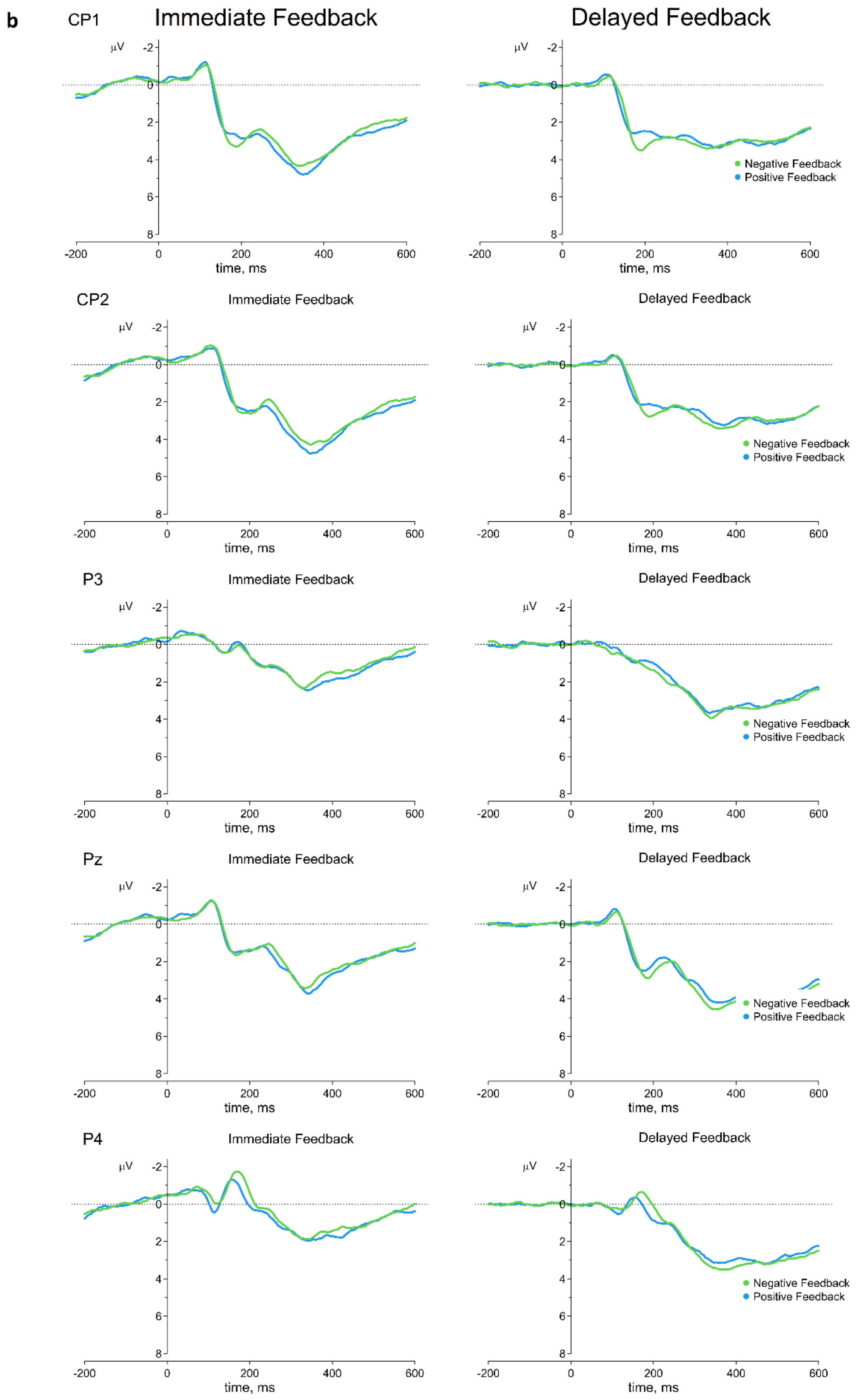
b
CP1
Immediate Feedback
Delayed Feedback
μV
-2
0
2
4
6
8
-200
0
200
400
600
time, ms
Negative Feedback
Positive Feedback
CP2
P3
Pz
P4

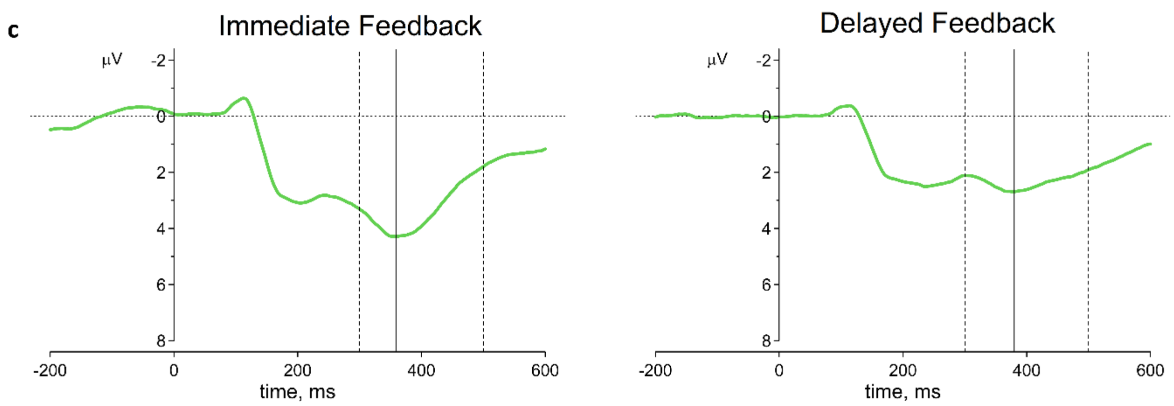

Supplementary Fig. S1 Grand-averaged ERPs. a The grand-averaged feedback-locked waveforms are shown separately for the immediate and delayed feedback timing condition and separately for trials with positive and negative feedback for each electrode of the frontocentral and **b** parietal cluster of electrodes considered in the analyses. In **c**, the grand average across all electrodes from the frontocentral and parietal cluster and across positive and negative feedback is shown, but separately for immediate and delayed feedback, illustrating the quantification of the P300. Dashed lines show the time window in which the P300 was quantified. The identified peak latency is shown with the solid line.

S2. Comparison of result patterns between models including prediction errors (PEs) vs fixed reward probabilities as predictors

To explore similarities and differences between the model-derived trial-level PEs (estimated for each participant individually, as described in detail in the Method section “Computational models to determine trial-by-trial PEs”) and the fixed reward probabilities of the stimuli (i.e. 0, 20, 40, 60, 80%) as predictors for the ERP amplitude data, we repeated the linear mixed-effects model analyses described in the manuscript with the objective reward probabilities instead of the model-derived PEs. All other parameters remained the same (analysis code including output can be found here: https://github.com/coweb101/fbdelaype).

For the FRN/RewP data, no effects in association with the objective reward probabilities were significant (all *p*s > .208). However, for the P300 data, significant effects of the objective reward probabilities in association with the other tested factors emerged. More specifically, while the main effect of Reward Probability on P300 amplitudes, as opposed to the main effect of the model-derived PE, was not significant ($p = .075$), the interaction between Reward Probability and Valence was significant, $b = 0.71$, $df = 18.74$, $t = 3.28$, $p = .004$. Similar as seen for the significant two-way interaction between the model-derived PE and Valence, follow-up simple slope analyses showed that the effect of Reward Probability is only significant for positive, $b = -1.28$, $z = -2.68$, $p = .015$, but not negative feedback, $b = 0.15$, $z = 0.68$, $p = 1.000$.

This relationship between P300, Reward Probability and Feedback Valence was further moderated by Feedback Timing, which was not seen for the model-derived PEs. More specifically, not only the two-way interaction between Feedback Timing and Reward Probability, ($b = -0.21$, $df = 113662.86$, $t = -2.41$, $p = .016$), but also the three-way interaction between these two factors and Valence ($b = -0.19$, $df = 106800.08$, $t = -2.24$, $p = .025$) was significant. Follow-up simple slope analyses indicated that for immediate as well as delayed feedback Reward Probability modulated amplitudes in response to positive but not negative

feedback (slope of Reward Probability for immediate positive feedback: $b = -1.27$, $z = -2.54$, $p = .044$, for immediate negative feedback: $b = 0.54$, $z = 2.29$, $p = .087$, slope for delayed positive feedback: $b = -1.30$, $z = -2.59$, $p = .038$, for delayed negative feedback: $b = -0.25$, $z = -1.07$, $p = 1.000$). Amplitudes in response to positive feedback were thereby larger (i.e. more positive) for stimuli with lower reward probabilities, i.e. for the stimuli for which rewards were less likely and therefore more likely to be unexpected. As can also be seen in Figure S2a, the direction of the effect of Reward Probability differed for immediate negative feedback compared to immediate positive and delayed positive and negative feedback, which was confirmed by follow-up pairwise comparisons (all *p*s $< .001$) and which has likely driven the three-way interaction.

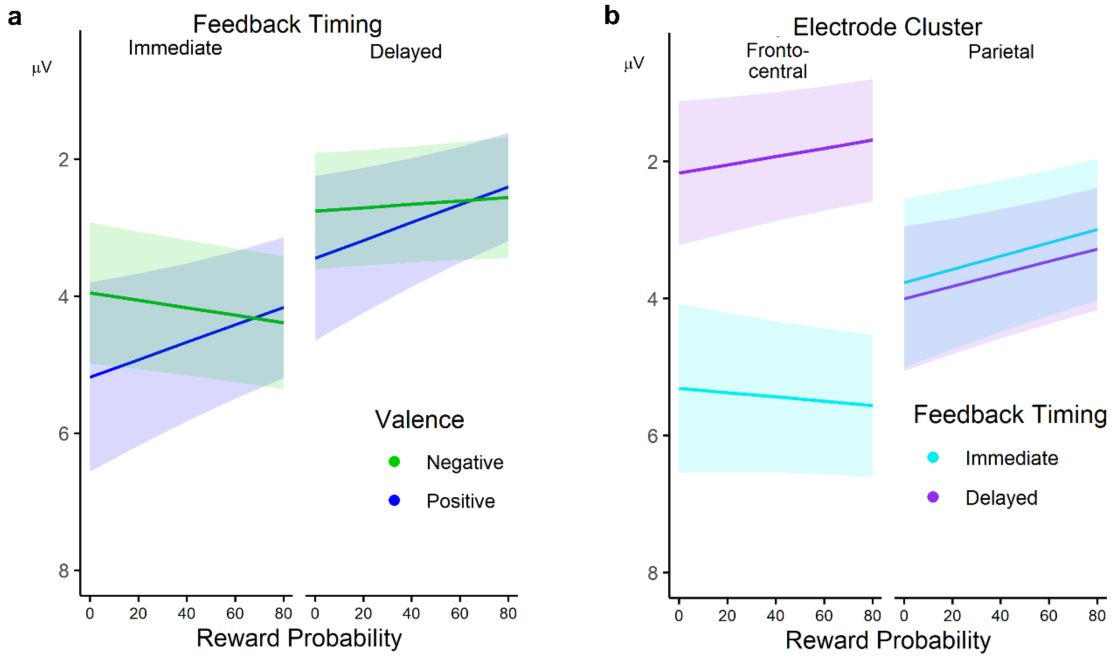

Supplementary Fig. S2 Effects of Fixed Reward Probabilities on P300 amplitudes. a Model-estimated marginal effects illustrating the interaction between the fixed effects of Feedback Valence, Feedback Timing, and Reward Probability, and **b** the interaction between the fixed effects of Feedback Timing, Electrode Cluster, and Reward Probability. Shaded areas represent 95% confidence intervals. Please note that the depicted marginal effects in **a** extrapolate to the impossible value 0 (reward probability) for positive feedback, which cannot be interpreted.

Furthermore, the two-way interaction between Reward Probability and Frontality ($b = 0.40$, $df = 116871.45$, $t = 4.72$, $p < .001$) as well as the three-way interaction between these

two factors and Feedback Timing (*b* = -0.25, *df* = 116871.45, *t* = -2.95, *p* = .003) was significant. Follow-up simple slope analyses indicated that Reward Probability only modulated amplitudes at parietal but not frontocentral electrode sites, similarly for immediate and delayed feedback (slope of Reward Probability (1) for immediate feedback at frontocentral electrodes: *b* = 0.28, *z* = 0.83, *p* = 1.000, (2) for immediate feedback at parietal electrodes: *b* = -1.01, *z* = -3.01, *p* = .010, (3) for delayed feedback at frontocentral electrodes: *b* = -0.63, *z* = -1.86, *p* = .249, (4) for delayed feedback at parietal electrodes: *b* = -0.92, *z* = -2.75, *p* = .024; see Fig. S2b). Since this parietal effect was not further modulated by Valence, it appears to be an effect of surprise rather than an encoding of positive or negative prediction errors (i.e. better-than-expected vs. worse-than-expected). Note that both interactions were not significant with the model-derived PEs. For the model-derived PEs, we found similar modulations in the P300 data from the frontocentral as well as the parietal electrode cluster, which interacted with Valence.

The pattern of the interaction found here, i.e. between Feedback Timing, Reward Probability, and Frontality (Fig. S2b), strongly resembles the three-way interaction between Feedback Timing, Feedback Valence, and Frontality found in the analysis with the model-derived PEs (see Fig. 3b). There we found a larger amplitude difference between immediate and delayed feedback for the frontocentral compared to the parietal electrode cluster, which is also apparent in the left panel of Fig. S2b. In this regard, it is important to note that the fixed reward probabilities are inherently confounded with feedback valence, i.e. there is a larger number of trials with positive feedback for stimuli with higher reward probabilities and vice versa. For the model-derived PEs, which trace the learning process of reward probabilities, this confound is less strong as we assumed equal stimulus values, and thus equal PEs, for the five stimuli in the first trial, which are then iteratively updated. That means, only with increasing trials (about 100), model-derived (absolute) PE values accumulate with a certain feedback valence and not, as is the case for the fixed probabilities, across all trials. Hence, it

can be assumed that effects of Reward Probability and Valence more strongly overlap than those of PE and Valence.

In summary, in contrast to the analysis with the model-derived trial-level PE, our analysis with fixed reward probabilities provides no evidence for a modulation of FRN/RewP amplitudes. For the P300, the analysis revealed a modulation by Reward Probability of amplitudes in response to positive feedback similarly as seen for the model-derived PEs. However, a valence-independent effect of Reward Probability on the P300 seems to be mainly reflected in responses at parietal but not frontocentral electrode sites, reflecting a different pattern as for the relationship between model-derived PEs and the P300.
